# Evaluation of Worksite Wellness Nutrition and Physical Activity Programs and Their Subsequent Impact on Participants' Body Composition

**DOI:** 10.1155/2018/1035871

**Published:** 2018-12-03

**Authors:** Victoria Sandercock, Jeanette Andrade

**Affiliations:** ^1^School of Family and Consumer Sciences, Eastern Illinois University, Charleston 61920, USA; ^2^Food Science and Human Nutrition, University of Florida, Gainesville, FL 32611, USA

## Abstract

**Background:**

Adult obesity is globally recognized as a public health concern. As adults spend most of their weekdays at work, worksite wellness programs may include topics of nutrition education and physical activity to improve an employee's body composition. However, results are inconsistent with the impact they have on employees' body composition.

**Objective:**

The purpose of this systematic review was to evaluate worksite wellness nutrition and physical activity programs and their subsequent impact on participants' body composition.

**Methods:**

Extraction of articles was completed through 4 databases: PubMed, CINAHL, SCOPUS, and PsycINFO using keywords such as “nutrition and physical activity interventions/programs” and “weight.” A 9-point inclusion criterion was established. Evaluation of the articles was assessed using the Academy of Nutrition and Dietetics Evidence-Based Manual.

**Results:**

A total of 962 articles were identified. Twenty-three met the inclusion criterion. Seventeen studies resulted in a change in body composition (e.g., decreased BMI (kg/m^2^), waist circumference, and body fat percentage), and six studies did not show any changes. Programs that had professionals frequently interact with participants, regardless if the interactions were done daily, weekly, or monthly, led to a change in body composition. Additionally, programs that incorporated a motivation theory and provided content relevant to participants' needs resulted in a change in body composition.

**Conclusion:**

Evidence supports that future worksite wellness programs that are designed using a motivational theory and content that is created relevant to participants' needs and that has frequent interactions with participants may result in a change in body composition.

## 1. Introduction

The global rise in obesity has deemed it a public health crisis. In 2016, the World Health Organization reported that globally over 1.9 billion adults, aged 18 and older, (39% of men and 40% of women), were considered overweight (BMI 25–29.9 kg/m^2^). Of those, 650 million (11% of men and 15% of women) were considered obese (BMI ≥ 30 kg/m^2^) [1]. Adult obesity contributes to 7 out of 10 deaths per year [2–4] and leads to several chronic health conditions such as cardiovascular disease (hypertension, stroke, and atherosclerosis), certain types of cancers (breast, colon, and prostate), and type II diabetes [2–9].

Adult obesity rates continue to rise for a number of reasons such as adults consuming less than the required amounts of whole grains, fresh fruits, and vegetables while increasing their consumption of high saturated fat and sodium foods [10–12] and reducing their physical activity [2, 4, 13–15]. Considering that employees spend an average of 8.0 hours working at their jobs compared to an average of 3.1 hours working at home [16], a worksite wellness program may be an ideal strategy to combat this rise in obesity.

Since the 1630s, organizations have implemented occupational programs for a variety of reasons such as for the prevention of injuries or to enhance employee performance [17–19]. In the mid-1970s, worksite wellness programs emerged to focus on the health of the employees [18, 19]. These worksite wellness programs are critical to have in place as employers are combating the number of sick days and productivity lost to employees who have chronic conditions such as obesity [14, 15, 18]. Healthcare costs associated with obesity are estimated to be about 190.2 billion dollars per year [20] with employers spending about 4.3 billion dollars per year due to employee-related absenteeism [20, 21] and another 506 million dollars per year due to lost productivity [22]. A 2013 report indicated that more than 50% of organizations are offering worksite wellness programs to enhance employees' health. These programs may include nutrition education, fitness, and weight management practices [23].

For worksite wellness programs to be effective in reducing the prevalence of obesity, physical activity and nutrition education should be incorporated within these programs [15, 24, 25]. Evidence has shown that regular physical activity (150 minutes per week) and consuming a nutritious diet (e.g., whole grains, fresh fruits, and vegetables) aid in weight reduction [26–29]. Therefore, common components included within these programs are health/risk assessments, exercising, and educational sessions geared toward nutrition and overall wellness [30–34]. Results from studies demonstrated that participants achieved program goals such as changing their body composition (e.g., decreasing body fat percentage and body mass index (BMI kg/m^2^)) [35–37]. Beyond the incorporation of physical activity and nutrition education, other factors included in the design of these programs that led to a change in body composition were the inclusion of a motivational theory, length of the program, and intensity of interaction. For instance, programs that were 12 months or longer resulted in decreased body weight. This may be because a change in behavior takes at least six months for it to be sustained [38]. An individual is less likely to carry on with a behavior change if they do not have ample opportunities within an extended period of time to restructure choices in regard to physical activity and eating nutritiously throughout daily living [27, 29, 39]. Plus, if one is motivated either internally or externally, this could help to integrate the program goals into everyday practice [39–42] to potentially change his or her body composition. However, results from worksite wellness programs that incorporated these components were inconsistent with changes in an individual's body composition [32, 43, 44]. Therefore, the purpose of this systematic review was to evaluate worksite wellness nutrition and physical activity programs and their subsequent impact on participants' body composition.

## 2. Materials and Methods

### 2.1. Search Strategy

This review was conducted by two independent researchers from Eastern Illinois University using the Preferred Reporting Items for Systematic review and Meta-analysis (PRISMA) [45]. Identification of studies followed a three-step process: (i) search, (ii) distillation, and (iii) independent review [46]. To assess the quality of the identified articles, the researchers used the Quality Criteria Checklist from the Academy of Nutrition and Dietetics (AND) Evidence Analysis Manual [46, 47]. No IRB approval was acquired as no humans or animals were involved in this study.

In the first phase, search, the researchers used four databases: PubaMed, CINAHL, SCOPUS, and PsycINFO to identify these articles [46]. The key search terms, in various combinations, were used to identify articles: “adults, 18 years and older,” “worksite wellness/employee wellness/organization,” “nutrition/dietary/nutritious,” “physical activity/exercise,” “interventions/programs,” “overweight/obesity,” “body composition/weight percentage,” and “body fat/weight/weight loss.” A language restriction was applied to include publications written in English. Also, a time restriction for the literature published between January 1, 1980, and November 1, 2017, was applied. The reference list of the retrieved articles was searched to identify other relevant manuscripts for this review. Literature searches were combined into EPPI-Reviewer 4 [48], a software to assist in screening and removing duplicate articles [46].

### 2.2. Article Screening

The second phase of the systematic review process was the distillation phase. This involved one researcher reading through the titles and abstracts to identify articles that met the following nine inclusion criterion [46]: studies (1) were published peer-reviewed; (2) were published between January 1, 1980 to November 1, 2017; (3) included experimental studies; (4) included participants over 18 years of age; (5) included participants that were overweight (BMI ≥ 25 kg/m^2^) and/or obese (BMI ≥ 30 kg/m^2^); (6) took place at a worksite; (7) had intervention components that included physical activity and nutrition education; (8) involved interventions that lasted 12 months or longer and (9) had outcome measurements that included body composition (BMI (kg/m^2^), waist circumference, and weight). Articles were excluded if they did not meet the above nine criteria.

The third phase of the systematic review process was the independent review of articles. In this phase, the two researchers independently read all remaining articles to determine the ones that met the inclusion criterion. After each reviewer independently identified the articles to keep, if discrepancies existed, a discussion took place until reviewers were satisfied with including or excluding the articles [46]. After this third phase, the remaining articles were critically evaluated for relevance and validity via the Quality Criteria Checklist from the Academy of Nutrition and Dietetics (AND) Evidence Analysis Manual [47]. Relevance determines a study's usefulness to the nutrition profession and is defined by four questions. If responses to all four questions were yes, the reviewers then proceeded to the validation questions; otherwise, the article was removed from this systematic review.

For validity, 10 criteria questions were used to determine the quality of these studies; a thorough discussion of each criterion is found within the AND Evidence Analysis Manual [47]. Each component within the validity portion of the analysis manual was answered with a yes, no, unclear, or not applicable. An article was determined of high quality (+) if responses to validity criterion 2, 3, 6, and 7 were yes with an additional yes from another criterion. An article was determined low quality (−) and subsequently removed from further analysis if the responses to criterion 2, 3, 6, and 7 and two additional criterion were no. An article was determined neutral (Ө) if responses to the validity criteria 2, 3, 6, and 7 was no or unclear.

After the two researchers independently evaluated the quality of the articles, interrater reliability was determined using quadratic weighted Cohen's kappa statistic [46, 49]. A quadratic weighted Cohen's kappa was selected to account for the degree of disagreement among the reviewers [46]. Kappa results were interpreted as follows: values ≤0 indicate no agreement, 0.01–0.20 indicates none to a slight agreement, 0.21–0.40 indicates fair agreement, 0.41–0.60 indicates moderate agreement, 0.61–0.80 indicates substantial agreement, and 0.81–1.00 indicates almost perfect agreement [46, 49].

### 2.3. Data Extraction

Table 1 was constructed and organized by the researchers to compare the data extracted from each article to be included in this systematic review. The data extracted included the first author's last name, publication date, location, research design, study duration, participants, design/intervention groups, theory/framework, intervention description, evaluation measures, and findings (Table 1).

## 3. Results

### 3.1. Article Selection

**Figure 1:**
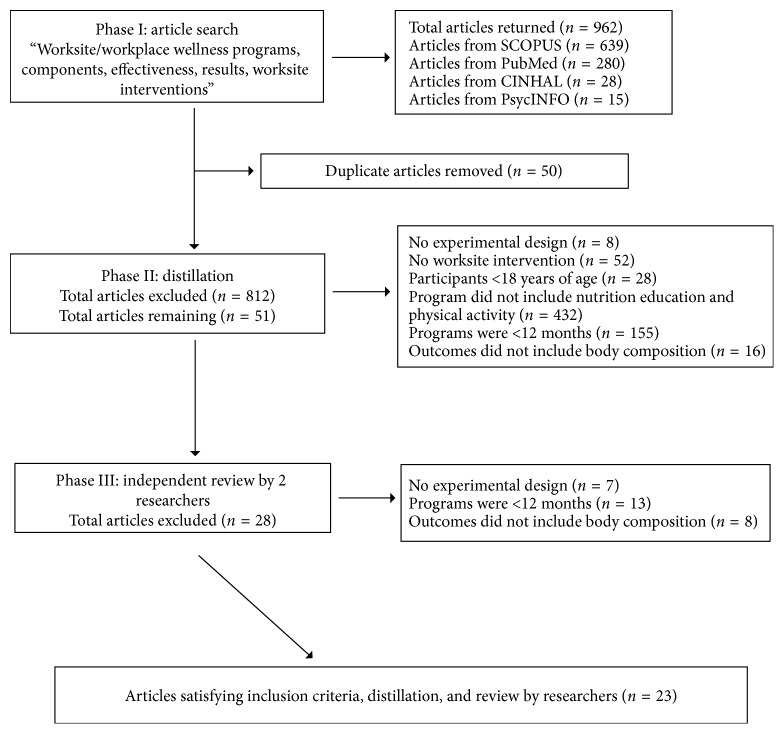
Article extraction.

A total of 962 articles were identified from the first search phase: PubMed (*n*=280), CINAHL (*n*=28), SCOPUS (*n*=639), and PsycINFO (*n*=15) databases. In the second, or distillation phase, 150 articles were found to be duplicates and therefore removed. Then, using a 9-point inclusion checklist, one researcher reviewed the remaining 812 articles. From this initial review, 761 articles were removed because they did not have an experimental design (*n*=78), did not include a worksite program (*n*=52), included participants younger than 18 years of age (*n*=28), did not include nutrition education and physical activity components (*n*=432), were less than twelve months in duration (*n*=155), and outcomes did not include an aspect of body composition (*n*=16). After phase two, 51 articles remained and were independently reviewed by two researchers. In this final phase, 28 articles were excluded because they did not have an experimental design (*n*=7), were less than twelve months in duration (*n*=13), and outcomes did not include an aspect of body composition (*n*=8) (Figure 1).

From the 23 articles that remained, the researchers assessed their quality. These studies were deemed high quality. None of the studies was rated as low quality. Unbiased endpoint assessment (i.e., not clearly stating if researchers were blinded), calculation of outside factors that could impact the results (i.e., dietary intake and physical activity behaviors in the home), variability in measurement techniques of body composition, and lost to follow-up received the lowest ratings across all items irrespective of study design. The overall kappa score from the researchers was 0.76, which demonstrates substantial agreement [49] (Table 2).

### 3.2. Study Range and Characteristics

The twenty-three studies included a total of 41,867 participants, ranging between 45 and 8,030 participants, with a mean of 1,819 participants [10, 31–37, 43, 44, 50–62]. Four studies did not report the age range of participants, but three studies included participants aged 18 years and older [10, 30, 50] and one study included participants aged 21 years and older [53]. The designs of these studies were randomized controlled trials (*n*=14) [10, 31, 34–37, 43, 44, 51–53, 56, 58, 62], prospective cohorts (*n*=5) [54, 55, 59–61], pretest/posttest (*n*=2) [32, 57], and quasi-experimental design (*n*=2) [33, 50]. The shortest duration of the interventions lasted one year [31, 35, 36, 43, 54, 56, 57, 60, 63], and the longest intervention lasted eight years [33].

### 3.3. Synthesis of the Intervention Results

Of the twenty-three studies, six studies offered monthly educational sessions [33, 35, 43, 55, 57, 58], three offered weekly educational sessions [31, 34, 36], two offered a one-time educational session [37, 62], and two offered an unspecified amount of educational sessions [32, 44]. These educational sessions included topics such as nutrition, physical activity, and health behaviors. Studies offered telephone health coaching throughout the intervention (*n*=3) [32, 54, 60] or in-person health coaching (*n*=1) [59], health screenings (*n*=5) [33, 51, 57, 61, 62], and provided magazines (*n*=1) [51] or posters (*n*=5) [32, 50, 52, 53, 56].

Specific physical activity components offered within some of the interventions aside from physical activity education, training, and encouragement included pedometer step counting [43], voluntary participation in sports [57], and on-site fitness facilities for employees [34, 53, 56].

Of the twenty-three studies, four of them incorporated incentives into the intervention [10, 33, 59, 61]. Incentives included being entered into a $250 cash prize lottery for completing a health screening [63] and redeeming healthy behavior points for cash prizes for logging behaviors such as exercising, being a nonsmoker or quitting, reducing or maintaining optimal blood pressure, cholesterol, and/or body fat levels, having routine preventative exams, and engaging in various educational activities on healthy lifestyle topics [33, 61].

Additionally, studies included some form of a theory or framework to guide the intervention. These included a social-based theory: Social Cognitive theory (*n*=1) [62] or Social Ecological Model (*n*=2) [35, 37]; a self-driving theory: Motivational Interviewing (*n*=1) [59]; a behavioral change theory: Cognitive Behavioral Training (*n*=1) [31] and Behavioral Change Framework (*n*=1) [55]; or a combination of theories: Social Support Model, Social Cognitive Theory, and Stages of Change Trans-Theoretical [51] and the Health Belief Model, Motivational Interviewing, and Stages of Change Trans-Theoretical [60]. Fifteen of the articles did not specify a certain theory but included elements such as a voluntary participation and incentives to encourage a self-motivation component within the intervention.

Studies varied in the measurement techniques for determining body composition. For determination of BMI, calibrated electronic/digital scales and a stadiometer were used (*n*=12) [10, 31, 33, 35, 36, 43, 44, 51–53, 56, 57] or had participants self-report their height and weight (*n*=1) [54]. For determination of body fat, a bioelectrical impedance analyzer (*n*=4) [31, 33, 43, 59] or skinfold calipers (*n*=2) were used [57, 61]. For determination of waist circumference, flexible tape measures were used (*n*=4) [31, 36, 43, 57]. However, there were research studies that indicated biometric measurements such as weight and height were taken, but did not report the device(s) used (*n*=8) [32, 34, 37, 50, 55, 58, 59, 62]. Seven studies provided specifics on measurements [31, 35, 44, 51–53, 56, 61]. Measurements were primarily taken at baseline and postintervention with a few measurements taken at certain time points of the intervention (e.g., baseline, 12 months, and 24 months) [32, 33, 37, 44, 50, 52, 55, 56, 58, 59, 61, 62].

Results from studies showed statistically significant changes in body composition (e.g., decreased BMI, body fat percentage, and waist circumference) [10, 33, 35–37, 52, 54, 56–61]. Even though body composition changed in other studies, these findings were not statistically significant [31, 50, 53]. Six of the interventions did not show any changes in body composition [32, 34, 43, 44, 51, 62], and one showed an increase in BMI [55].

## 4. Discussion

Twenty-three studies were included in this systematic review to evaluate worksite wellness nutrition and physical activity programs and their subsequent impact on participants' body composition. Overall, results from this systematic review showed inconsistencies with the effect worksite wellness programs had on participants' body composition. In which, 13 studies resulted in significant changes in body composition (e.g., decreased BMI (kg/m^2^), body fat percentage, and waist circumference), three resulted in nonsignificant changes in body composition (e.g., slightly decreased BMI (kg/m^2^), body fat percentage, and waist circumference), six resulted in no changes in body composition and one showed an increase in BMI. Interventions that lasted for 48 or 96 months consistently demonstrated significant changes in body composition compared to those interventions that lasted between 12 and 36 months. Furthermore, regardless of the length of the intervention, participants who were able to interact with others on a consistent basis (e.g., interactive websites, group discussions, or one-on-one health coaching) were more likely to change their body composition (e.g., decreased BMI (kg/m^2^), body fat percentage, and waist circumference). However, there were inconsistencies with the incorporation of physical activity and change in participants' body composition as well as measurement techniques to analyze body composition.

Further analysis of these programs showed those that used a self-motivation theory (Motivational Interviewing) resulted in a greater change in body composition (e.g., decreased BMI (kg/m^2^), body fat percentage, and waist circumference) compared to those programs that did not use this type of theory. Motivational Interviewing is described as a direct, client-centered counseling style to initiate or derive a behavior change by means of aiding individuals and educating to resolve uncertainty [64]. Two of the studies within the review specifically utilized Motivational Interviewing as the framework for the interventions [59, 61]. Merrill and Merill [59] conducted a 4-year workplace program that was designed based on Motivational Interviewing and included health courses and personal telephone coaching. Participants (*n*=10,342) were split into 4 groups: year 1 (*n*=1,814), year 2 (*n*=2,777), year 3 (*n*=2,739), and year 4 (*n*=3,012). Results showed that all groups were successful in decreasing BMI and body fat percentage. Conclusions indicated that engaging and motivating participants to change their behaviors led to positive weight outcomes. In accordance with this study, several other studies demonstrated that Motivational Interviewing engages participants and ultimately changes aspects of their behaviors to improve health outcomes [65–67]. Kouwenhoven-Pasmooij and colleagues (2018) [67] showed that by incorporating Motivational Interviewing within a health-risk assessment class, participants (*n*=274) were more motivated and engaged and increased their participation in health-promotion activities compared to those participants (*n*=217) that were not. The researchers concluded that Motivational Interviewing should be incorporated into classes to further engage participants in health-promoting activities. Even though modest changes in body composition were seen in the studies that directly used a theory, the design, methods, and frequency of providing information to participants may have also led to changes in body composition.

The educational content presented in these interventions focused on increasing healthy eating habits, physical activity, and healthy behaviors presented in a variety of methods such as emails, face to face, telephone calls, interactive websites, newsletters, posters, or a combination of methods. Participants had a reduction in body composition if the educational content was based on their knowledge or the content's messaging was tailored to their knowledge level. Designing content that is tailored to an individual's knowledge and attitudes may increase his or her self-motivation to change health behaviors [68, 69]. However, results from a few studies, in which tailored messaging were incorporated within the interventions, did not show a change in body composition [32, 51, 70]. This may be because the messages were not designed based on participants' knowledge and attitudes, participants were not motivated, or it is not known whether the frequency of these messages was conveyed to the participants. The frequency of researchers interacting with participants ranged from daily [63] to monthly [33, 43, 57, 59, 70] and for a minimum interaction time of 15 minutes [54] to a maximum of one hour [31, 34–36, 58]. If participants received frequent interaction, regardless of the length of time and the method, there were changes in the participants' body composition. Other studies, including systematic reviews, reported that participants who had frequent interactions with health professionals reduced their body weight significantly more compared to those who had limited or no interactions [71–75]. Receiving continuous feedback or support from health professionals may enhance participants' motivation and vigilance to maintain their lifestyle behaviors. However, this is not always the case as a study [34] showed. Participants in the intervention group (*n*=377) were exposed to weekly 30- or 60-minute nutrition educational sessions over 12 months. However, there were no changes in BMI compared to the control group. This may have been because participants were not motivated or provided with sufficient support. Thus, future worksite wellness programs should consider the depth of the educational content and the frequency of interaction for participants to remain motivated to adhere to the program and ultimately affect their body composition.

Furthermore, there were inconsistencies with the impact physical activity had on participants' body composition. Studies used various devices such as pedometers and accelerometers to increase physical activity among participants in these worksite interventions [31, 43, 55]. Pedometers measure steps and total physical activity but not intensity, while accelerometers measure frequency but not the type of exercise [76]. Neither can provide adequate information on the duration, frequency, and intensity of the activity, which would provide a more reliable measure of energy expenditure [77]. Aparicio-Ugarriza and colleagues [77] conducted a review of the literature to identify a method to better assist with tracking physical activity. Results showed that all measurement devices developed for tracking physical activity in the field contained limitations such as the inability to measure intensity, frequency, and type of physical activity. To fully understand the impact physical activity has on body composition, further development needs to be conducted on these devices that can obtain information on intensity, duration, and frequency.

The main outcomes of this review showed a change in participants' body composition; however, the tools used to measure body composition fluctuated. Moreover, these studies used simple, quick, and noninvasive tools to measure body composition due to the number of participants and the location of these studies (e.g., businesses). Twelve studies indicated the use of a calibrated scale to measure body weight and a stadiometer to measure height to determine participants' BMI. Only six of those studies indicated the techniques used to take these measurements (e.g., no socks or shoes, measured to the nearest kg). The accuracy of these scales, though, may not have been adequate. A report that evaluated 223 scales in health-care clinics and fitness and weight loss centers showed that increased weight led to decreased precision. Moreover, more than 25% of the scales were off by more than 0.9 kg, and 15% were off by more than 2 kg [78]. Additionally, the condition of the scales, surface (e.g., rug and concrete), and calibration history was not reported, which may have led to imprecise body weight. Furthermore, one study collected weight and height based on self-reported data, which may have been inaccurate as participants may have underreported their weight and over reported their height or had recall bias [79–81]. Additionally, the weight of the individual cannot determine the percentage of body fat or muscle mass, which may produce erroneous classifications of BMI, thus reporting BMI should be with caution [82]. For example, a bodybuilder with 12% body fat may potentially be characterized as overweight due to his weight compared to his height. Therefore, using other tools to define body composition may be more effective. Four studies used a bioelectrical impedance analyzer (BIA), and two studies used skinfold calipers to measure body fat. The studies which measured body fat with a BIA did not specify the body parts measured (e.g., hand to hand, foot to foot, or hand to foot), the conditions (e.g., fasting/hydration status, exercise status, and phase of menstrual cycle), nor the calibration techniques, which may have affected the measurement accuracy [83–85]. Also, BIAs are more accurate in a BMI range of 17–34 kg/m^2^, and in the included studies, some participants had a BMI > 34 kg/m^2^ [85, 86]. Congruent with BIA measurement issues, skinfold measurements may also be inaccurate due to age, gender, and variability of fat distribution [87, 88]. Additionally, limited information was provided about calibration and the number of times measurements were repeated [89]. Finally, four studies used a tape measurer for determination of waist circumference. As with the other measurement tools, limited information was provided about the methods used to obtain the measurements (e.g., relax stance, normal expiration, and repeated measures). In addition, inaccuracies of measurements may have been obtained especially if the tape could not lay flat against the skin due to deposits of fat or the marked curvature near the iliac crest [90, 91]. From the 23 studies, six used a variety of body composition measurement tools and techniques to reduce measurement inaccuracies from using only one tool. Therefore, future studies should include the exact techniques they are using to measure one's weight as well as consider multiple tools to reduce any potential measurement inaccuracies.

### 4.1. Limitation

A limitation of this systematic review was that the search focused on nutrition education and physical activity in worksite programs that were long-term, thus limiting the potential number of articles. Second, while some of the studies reviewed showed positive results, the methods of collecting body composition data were inconsistent. The methods to measure body composition included body fat percentage calculation, body weight measurement, BMI calculation, and waist circumference measurement. To improve this limitation, inclusion criteria could have specified body measurement techniques. This would have created consistencies across the studies but would have also potentially limited the number of studies that would have been able to be included within the review. Another limitation was that the studies did not specifically measure baseline diet quality and did not keep adequate records of food intake throughout the studies. This could have caused variation in findings between participants. To better analyze the diet quality, the researchers could have included a quality analysis of diet at baseline as well as assigned a diet recall for participants to record throughout the studies. It is known that physical activity can be effective for reducing weight, but the combination of both diet and exercise has been proven to be the most effective means of weight loss [92, 93]. Because of this, it would have been beneficial to take a closer look at the dietary behaviors of participants. If participants were practicing nutritious eating behaviors while also increasing physical activity, weight loss would be more likely to occur [94].

## 5. Conclusion

Worksite wellness programs that were designed using motivational theories, content was created based on participants' needs and participants had frequent interactions with health professionals, resulted in a change in participants' body composition. Future research would be beneficial for the continued analysis of worksite wellness program components. Breaking programs apart into the basic components could help to identify what is effective in not only changing body composition but also decreasing risk factors for chronic diseases such as type 2 diabetes and heart disease. While self-motivation theories and intensive educational sessions have been identified as effective components, there are more elements of programs to explore such as health assessments, health marketing, and health campaigns. If the most effective and beneficial components of worksite programs could be identified, all future worksite wellness programs could be modeled using only favorable elements to warrant positive results.

## Figures and Tables

**Table 1:** Summary of articles included within the systematic review (*n*=23).

Author (year)	Location	Design/duration	Population/groups	Theory/wellness intervention	Evaluation measures	Measurement techniques	Results
Allen et al. (2012) [43]	USA	RCT^1^,12 months	Total *n*=55 Intervention *n*=26 Control *n*=29	No specific theory Intervention: monthly education sessions and pedometers Control: no intervention	BMI^2^, BF%^3^, WC^4^	Weight: calibrated electronic scale Height: stadiometer BF: BIA^5^ WC: tape measurer No further specifications	Intervention: insignificant decrease in BMI, BF%, or WC (*p* > 0.05) Control: WC increased (*p* < 0.05)

Almeida et al. (2015) [10]	USA	RCT,12 months	Total *n*=1,790 Intervention *n*=1001 Control *n*=789	No specific theory Intervention: daily email, incentives, education website Control: quarterly newsletter and 1-hour resource session	BW^6^, BMI	Weight: calibrated electronic scale Height: stadiometer No further specifications	Intervention: BW and BMI decreased (*p* < 0.05)

Baker Parker et al. (2010) [50]	USA	Quasi-experimental: 3 groups, 24 months	Total *n*=2,431 Intense *n*=1,520 Moderate *n*=382 Control *n*=529	No specific theory Intense intervention: health goals, prompts, and messages Moderate intervention: prompts and messages Control: no intervention	BMI	Weight and height were measured, but no further specifications	An insignificant decrease in BMI (*p* > 0.05)

Barham et al. (2011) [36]	USA	RCT,12 months	Total *n*=45 Intervention *n*=21 Control *n*=24	No specific theory Intervention: 12 1-hour weekly sessions; 1-hour monthly sessions Control: 3-month delay of intervention	BMI, WC	Weight: calibrated electronic scale Height: stadiometer WC: tape measurer. Measurements were taken by trained professionals, no further specifications	Intervention: decreased BMI (*p* < 0.001) and WC (*p*=0.004) in the first three months

Campbell et al. (2002) [51]	USA	RCT,18 months	Total *n*=660 Intervention *n*=362 Control *n*=298	Social cognitive theory, Stages of Change Trans-Theoretical framework, social support model Intervention: two personalized tailored “magazines,” Natural helpers Control: after 6 months, 1 magazine was provided	BMI	Weight: calibrated scale Height: tape measure Weight and height measured without shoes	No changes in BMI

Christensen et al. (2012) [31]	Denmark	RCT,12 months	Total *n*=98 Intervention *n*=54 Control *n*=44	Cognitive behavioral training Intervention: 1-hour weekly educational sessions Control: monthly 2-hour oral presentations	BW, BMI, BF%	Weight: calibrated scale. Measurements taken without socks and shoes, light clothing, 1 kg subtracted to compensate for clothing. Height: stadiometer Measurements taken without shoes BF: BIA. Measured by “standard” body frame and participant's age, height, and gender BF: tape measurer. Waist circumference measured over umbilicus. Hip circumference measured on the hip part that gave the greatest circumference	Intervention: BW decreased by 6 kg (*p* < 0.001) BMI decreased by 2.2 kg/m^2^ (*p* < 0.001) BF% decreased by 2.8% (*p* < 0.001)

Fernandez et al. (2015) [52]	USA	RCT: 10 groups,24 months	Total *n*=2,996 Intervention *n*=1,882 Control *n*=1,114	No specific theory Intervention: marketing at workplace, newsletters, interactive website Control: no intervention	BMI	Weight: calibrated electronic scale Height: stadiometer Measurements were taken in street clothes and without shoes	Intervention: BMI decreased by 0.54 kg/m^2^ (*p*=0.02) Control: BMI decreased by 0.12 kg/m^2^ (*p*=0.73)
French et al. (2010) [53]	USA	RCT, 18 months	Total *n*=696	No specific theory Intervention: healthy vending machine options, fitness facility, self-weighing competition, 2-day long health expos, monthly farmers markets Control: quarterly advisory group	BMI	Weight: calibrated electronic scale Height: stadiometer Measurements taken in street clothes and without shoes; 2 separate measurements taken and then averaged the values	Intervention: BMI decreased by −0.14 kg/m^2^ (*p* < 0.005)

Goetzel et al. (2014) [54]	USA	1 cohort group, 12 months	Total *n*=2,458	No specific theory Intervention: 15-minute individual telephone health coaching and online interactive wellness tools	BMI	Self-reported weight and height No specifications on how they trained participants to take these measurements	BMI decreased by 2.0 kg/m^2^ (*p* < 0.001)

Hochart et al. (2011) [32]	USA	Pretest, posttest: 13 groups, 36 months	Total *n*=8,030 Intervention *n*=4,230 Control *n*=3,800	No specific theory Intervention: telephone coaching, worksite or webinar education classes, online resources, and behavior change support tools Control: no intervention	BW, BMI	Weight and height measured, but no specifications on how these measurement techniques were taken	Intervention: Insignificant decreases in BW and BMI (*p* > 0.05)

LeCheminant et al. (2012) [55]	USA	1 cohort group 24 months	Total *n*=174	Behavioral change framework Intervention: 6, 3- to 8-week intervention campaigns	BMI	Weight and height measured, but no specifications on how these measurement techniques were taken	BMI increased (*p* < 0.05)

Lemon et al. (2014) [56]	USA	RCT, 24 months	Total *n*=782 Intervention *n*=446 Control *n*=336	Social ecological model Intervention: fitness facilities, healthy lunchroom options, elimination of SSB^7^, healthy prompts, print, and web-based materials Control: print and web-based materials only	BMI	Weight: calibrated electronic scale. Measured by trained staff, readings to the nearest 2/10th pound Height: stadiometer. Measured by trained staff, readings to the nearest 1/8th inch	Decreased BMI -0.48 kg/m^2^ (*p*=0.05)

Leyk et al. (2014) [57]	Germany	Pretest, posttest: 3 groups, 12 months	Total *n*=474 Nonactive *n*=129 Not very active *n*=209 Very active *n*=136	No specific theory Intervention: voluntary sport participation, sports-medicine exam, monthly lectures	BW, BMI, BF%, WC	Weight: calibrated electronic scale Height: stadiometer BF: caliper WC: tape measurer Sports-medicine specialist took the measurements, no specifications of how measurements were taken	Decrease in BW (*p*=0.002), BMI (*p* < 0.001), and WC (*p*=0.001) among men No significant differences among women

Linde et al. (2012) [44]	USA	RCT, 24 months	Total *n*=1,406 Intervention *n*=611 Control *n*=795	No specific theory Intervention: education classes Control: no intervention	BW, BMI	Weight: calibrated electronic scale. Trained team specialists measured to the nearest 0.1 kg wore light street clothes without shoes Height: stadiometer Trained team specialists measured to the nearest 0.1 cm	Intervention: no changes in BW and BMI
Mache et al. (2015) [34]	Germany	RCT, 12 months	Total *n*=675 Intervention *n*=377 Control *n*=298	No specific theory Intervention: weekly 30–60 minutes training sessions, healthy food and exercise demonstrations, and activities Control: no intervention	BMI	Weight and height measured but no further specifications	No changes in BMI

MacKinnon et al. (2010) [58]	USA	RCT: 3 groups, 48 months	Total *n*=599 MI *n*=202 TEAM *n*=234 Control *n*=163	No specific theory TEAM: 11–45 minute sessions in the first year and 6 booster sessions in the second year MI: 4 1-hour individual motivational interviewing sessions in the first year, and 2 meetings with a counselor and optional additional 6 hours of in-person or phone contact in the second year Control: no intervention	BMI	Weight and height measured but no further specifications	Decreased BMI for TEAM intervention at 1 year (*p*=0.06)

Merrill et al. (2014) [59]	USA	1 cohort group, 48 months	Year 1 *n*=1,814 Year 2 *n*=2,777 Year 3 *n*=2,739 Year 4 *n*=3,012	Motivational interviewing theory Intervention: monetary incentives, monthly newsletter, individual health coaching	BMI, BF%	BMI: weight and height measured but no further specifications BF%: body fat analyzer, but no further specifications	Over the 4-year period, decreased BMI (*p* < 0.05)

Merrill et al. (2010) [60]	USA	1 cohort group, 12 months	Total *n*=6,128	Healthy belief model, Trans-theoretical model of change, motivational interviewing Intervention: telephonic health coaching	BMI	Weight and height measured but no further specifications	Decreased BMI (*p* < 0.001)

Muto et al. (2001) [37]	Japan	RCT, 18 months	Total *n*=302 Intervention *n*=152 Control *n*=150	No specific theory Intervention: 4-day education program, individual counseling, group discussions Control: no intervention	BMI	Weight and height measured but no further specifications	Intervention: decreased BMI by 0.5 kg/m^2^ (*p* < 0.05)

Neville et al. (2011) [33]	USA	Quasi-experimental: 3 groups, 96 months	Total *n*=365 Group 1 *n*=108 Group 2 *n*=106 Group 3 *n*=151	No specific theory Intervention: monthly education sessions, incentives, health screenings Control: no intervention	BW, BMI, BF%	Weight: calibrated electronic scale Height: stadiometer BF: BIA Trained health educator staff members took measurements, but no further specifications	Decreased BMI for the highest risk group (group #1) (*p* < 0.05)

Poole et al. (2001) [61]	USA	1 cohort group, 48 months	Total *n*=304	No specific theory Intervention: annual health assessment, incentive system	BF%	Skinfold calipers. Measurements were taken at the chest, abdomen, and thigh for men and triceps, supra-ilium, and thigh for females	Decreased BF% (*p* < 0.05)

Robroek et al. (2012) [62]	Netherlands	RCT, 24 months	Total *n*=924 Intervention *n*=465 Control *n*=459	Social cognitive theory Intervention: physical health check, face-to-face advice, personal feedback, monthly emails Control: no intervention	BMI	Weight and height measured, no further specifications. In the follow-up measurement, height and weight were self-reported	No decreases in BMI (*p* > 0.05)
Salinardi et al. (2013) [35]	USA	RCT, 12 months	Total *n*=133 Intervention *n*=94 Control *n*=39	Social ecological model Intervention: 19 1-hour long education sessions Control: no intervention	BW	Weight: calibrated electronic scale Measurements were taken with light indoor *clothing* and measured to 0.05 kg Height: stadiometer, no further specifications	BW decreased by an average of 8 kg (*p* < 0.05)

*Note.* 1 = randomized control trial, 2 = body mass index, 3 = body fat percentage, 4 = waist circumference, 5 = bioelectrical impedance analyzer, 6 = body weight, and 7 = sugar-sweetened beverages.

**Table 2:** Quality of studies within the systematic review (*n*=23).

Author (Year)	Quality rating	Research question stated	Clear of selection bias	Comparable study groups	Withdraws discussed	Blinding use	Intervention described	Outcomes defined	Appropriate statistical analyses	Results support conclusions	No potential for funding bias
Allen et al. (2012) [43]	+	Y	Y	Y	N	N	Y	Y	Y	Y	Y
Almeida et al. (2015) [10]	+	Y	Y	Y	N	N	N	Y	Y	Y	Y
Baker Parker et al. (2010) [50]	+	Y	Y	Y	N	N	Y	Y	Y	Y	Y
Barham et al. (2011) [36]	+	Y	Y	Y	N	N	Y	Y	Y	Y	Y
Campbell et al. (2002) [51]	+	Y	Y	Y	N	N	Y	Y	Y	Y	Y
Christensen et al. (2012) [31]	+	Y	Y	Y	N	Y	Y	Y	Y	Y	Y
Fernandez et al. (2015) [52]	+	Y	Y	Y	N	N	Y	Y	Y	Y	Y
French et al. (2010) [53]	+	Y	Y	Y	N	N	Y	Y	Y	Y	Y
Goetzel et al. (2015) [54]	+	Y	Y	Y	N	N	Y	Y	Y	Y	Y
Hochart et al. (2011) [32]	+	Y	Y	Y	N	N	Y	Y	Y	Y	Y
LeCheminant et al. (2012) LeCheminant	+	Y	N	N/A	N	N	Y	Y	Y	Y	Y
Lemon et al. (2014) [56]	+	Y	Y	Y	N	N	Y	Y	Y	Y	Y
Leyk et al. (2014) [57]	+	Y	N	Y	Y	N	Y	Y	Y	Y	Y
Linde et al. (2012) [44]	+	Y	Y	Y	N	Y	Y	Y	Y	Y	Y
Mache et al. (2015) [34]	+	Y	Y	Y	N	N	Y	Y	Y	Y	Y
MacKinnon et al. (2010) [58]	+	Y	Y	Y	N	N	Y	Y	Y	Y	Y
Merrill et al. (2014) [59]	+	Y	Y	Y	N	N	Y	Y	Y	Y	Y
Merrill et al. (2010) [60]	+	Y	Y	Y	N	N	Y	Y	Y	Y	Y
Muto et al. (2001) [37]	+	Y	Y	Y	N	N	Y	Y	Y	Y	Y
Neville et al. (2011) [33]	+	Y	Y	N/A	Y	N	Y	Y	Y	Y	Y
Poole et al. (2001) [61]	+	Y	Y	Y	N	N	Y	Y	Y	Y	Y
Robroek et al. (2012) [62]	+	Y	Y	Y	N	Y	Y	Y	Y	Y	Y
Salinardi et al. (2013) [35]	+	Y	Y	Y	Y	N	Y	Y	Y	Y	Y

*Note.* Quality ratings: (+) = positive.
